# The Efficacy of Exercise in Reducing Depressive Symptoms among Cancer Survivors: A Meta-Analysis

**DOI:** 10.1371/journal.pone.0030955

**Published:** 2012-01-27

**Authors:** Justin C. Brown, Tania B. Huedo-Medina, Linda S. Pescatello, Stacey M. Ryan, Shannon M. Pescatello, Emily Moker, Jessica M. LaCroix, Rebecca A. Ferrer, Blair T. Johnson

**Affiliations:** 1 Center for Clinical Epidemiology and Biostatistics, Perelman School of Medicine, University of Pennsylvania, Philadelphia, Pennsylvania, United States of America; 2 Institute for Translational Medicine and Therapeutics, Perelman School of Medicine, University of Pennsylvania, Philadelphia, Pennsylvania, United States of America; 3 Center for Health, Intervention and Prevention, University of Connecticut, Storrs, Connecticut, United States of America; 4 Department of Physical Therapy, M.D. Anderson Cancer Center, Houston, Texas, United States of America; 5 Department of Psychology, Western New England College, Springfield, Massachusetts, United States of America; 6 Biobehavioral and Psychological Sciences, National Cancer Institute, Rockville, Maryland, United States of America; Universidad Europea de Madrid, Spain

## Abstract

**Introduction:**

The purpose of this meta-analysis was to examine the efficacy of exercise to reduce depressive symptoms among cancer survivors. In addition, we examined the extent to which exercise dose and clinical characteristics of cancer survivors influence the relationship between exercise and reductions in depressive symptoms.

**Methods:**

We conducted a systematic search identifying randomized controlled trials of exercise interventions among adult cancer survivors, examining depressive symptoms as an outcome. We calculated effect sizes for each study and performed weighted multiple regression moderator analysis.

**Results:**

We identified 40 exercise interventions including 2,929 cancer survivors. Diverse groups of cancer survivors were examined in seven exercise interventions; breast cancer survivors were examined in 26; prostate cancer, leukemia, and lymphoma were examined in two; and colorectal cancer in one. Cancer survivors who completed an exercise intervention reduced depression more than controls, *d*_+_ = −0.13 (95% CI: −0.26, −0.01). Increases in weekly volume of aerobic exercise reduced depressive symptoms in dose-response fashion (β = −0.24, p = 0.03), a pattern evident only in higher quality trials. Exercise reduced depressive symptoms most when exercise sessions were supervised (β = −0.26, p = 0.01) and when cancer survivors were between 47–62 yr (β = 0.27, p = 0.01).

**Conclusion:**

Exercise training provides a small overall reduction in depressive symptoms among cancer survivors but one that increased in dose-response fashion with weekly volume of aerobic exercise in high quality trials. Depressive symptoms were reduced to the greatest degree among breast cancer survivors, among cancer survivors aged between 47–62 yr, or when exercise sessions were supervised.

## Introduction

There are over 12 million cancer survivors in the US [1]. Nearly 100% of all cancer survivors experience psychological and physical symptoms and side effects related to cancer or cancer treatment [2]. Cancer survivors may experience fear of death, disease relapse, and body image changes [3] that can contribute to the depressive symptoms experienced by up to 60% of cancer survivors [4] compared to 7% of the general US population [5]. Depression is associated with chemotherapy noncompliance [6], [7] and reduced 5 yr survival rates [8], [9]. Therefore, appropriate management of depressive symptoms among cancer survivors is of clinical importance. Exercise is an effective non-pharmacological therapy to reduce depressive symptoms among those living with depression [10], with a moderate standardized mean reduction when compared to those who do not exercise. Exercise provides similar or larger reductions in depressive symptoms in an array of clinical populations including those living with chronic obstructive pulmonary disease [11], human immunodeficiency virus [12], and coronary artery disease [13].

Accumulating evidence suggests exercise training after diagnosis of cancer may improve functional capacity, muscular strength, quality of life, and reduce cancer-related fatigue [14]–[16], but the efficacy of exercise to reduce depressive symptoms is inconsistent [2]. Some studies have demonstrated moderate to large reductions in depressive symptoms as the result of exercise [17], [18], whereas others observe no such reductions [19], [20]. Although a previous meta-analysis quantified the heterogeneity of exercise interventions to reduce depressive symptoms among cancer survivors and reported a moderate to large amount of heterogeneity (*I* ^2^ = 55%–76%), it did not examine moderator variables that could explain the variability in results [16].

Therefore, this meta-analysis examined the efficacy of exercise to reduce depressive symptoms among cancer survivors, confirming a previous meta-analysis [16], and attempted to identify exercise prescription and clinical factors associated with the greatest reductions in depressive symptoms. Identification of characteristics moderating the magnitude of reduction in depressive symptoms may aid clinicians in prescribing tailored exercise interventions to manage depressive symptoms among cancer survivors.

## Methods

### Inclusion Criteria

Studies were identified on *a priori* criteria that included: (1) a randomized controlled design comparing an exercise intervention with a control group (i.e., no exercise program prescribed and instructions to maintain current activity levels or no exercise related information); (2) report of depression outcomes; and (3) adults diagnosed with any type of cancer, regardless of stage of diagnosis or type or stage of treatment. Exercise interventions occurring in any setting, with or without supervision, were eligible.

### Systematic Search

The databases PubMed, PsycINFO, CINAHL Plus, SPORTSdiscus, OregonPDF in Health and Performance, and ProQuest Theses and Dissertations were searched through Nov 18, 2010. We searched all databases using a Boolean search strategy [i.e., (cancer OR neoplas* OR tumor OR chemo* OR radiat* OR malign* OR carciniom*) AND (depress* OR anxiety OR anxious OR worried OR scared OR nervous OR cognitive OR biofeedback OR relaxation OR social support OR mind-body) AND (exercise OR physical activity OR aerobic OR cardiovascular OR resistance OR strength OR muscular OR flexibility OR walking OR program OR interval OR sport OR fitness OR performance OR movement OR stretching OR tai chi OR yoga OR dance OR body OR composition)]. Journals focusing on cancer survivorship (*Journal of Clinical Oncology, Breast Cancer Research and Treatment*, *Journal of Cancer Survivorship*, *Oncology Nursing Forum*), and the reference lists of included studies were also searched for additional reports.

### Coding and Reliability

We estimated the intensity of exercise using the compendium of metabolic equivalent units (METs), where 1 MET represents sitting quietly (3.5 ml O_2_·kg^-1^·min^-1^) and <3 METs, 3 to <6 METs, and ≥6 METs represent low, moderate, and vigorous intensity exercise, respectively [21]. We calculated the weekly volume of aerobic exercise as the product of minutes of daily exercise and frequency of exercise sessions per week (min·wk^−1^). We used the Physiotherapy Evidence Database scale (PEDro) to gauge methodological quality of the trials in terms of internal validity and statistical reporting [22]. Four independent, trained raters extracted information related to the study with high inter-rater reliability, mean Cohen's κ = 0.90, for categorical variables, and mean intra-class correlation *r* = 0.94 for continuous variables.

### Study Outcome and Effect Size Calculation

The studies assessed depressive symptoms among cancer survivors as a continuous outcome variable assessed as a component of a comprehensive psychological questionnaire with a depression subscale [23] or a questionnaire solely assessing depression levels [24]–[27]. To assess baseline levels of depressive symptoms on a common metric across depression questionnaires, we used a 0–100 scale, where ‘0’ implies absolutely no depressive symptoms, and ‘100’ implies the highest level of symptoms possible on a given scale. We used the standardized mean difference effect size (*d*) to quantify the difference in depression from baseline to follow-up between the exercise and control groups, correcting for small sample size bias [28], [29]. The effect size *d* denotes the difference between the mean depression values of the control and exercise groups, divided by the pooled standard deviation [30]; the sign of *d* values was set to be negative when the exercise group reduced depression more than the control group. The standardized *d* value can be interpreted as −0.20, −0.50, and −0.80, represent small, medium, and large reductions in depressive symptoms, respectively [31]. When trials included more than one exercise group (e.g., aerobic exercise and resistance exercise), we calculated multiple effect sizes. Sensitivity analysis examined the influence of a single study on the overall mean effect size of all trials by iteratively removing a single study and then re-estimating the overall mean effect with 95% confidence intervals [32]. We present overall mean effect sizes (*d_+_*) as both, fixed- and random-effects estimates.

We used Stata 11.1 (StataCorp, College Station, TX) with macros developed for meta-analysis [33] to perform all statistical analyses. Begg's test [34] (*z* = −1.67, p = 0.10), Egger's test [35] (*t* = −0.12, p = 0.90), and the trim-and-fill method [36] identified no asymmetries in the effect size distribution suggestive of publication bias. Potential heterogeneity or between-study variance was calculated as *Q* and *I* ^2^ (and 95% CI) [37], [38]. The *Q* statistic follows an approximate *χ* ^2^ distribution with *k*-1 degrees of freedom, where *k* is the number of studies included in the meta-analysis [38]. The *Q* statistic can be standardized to *I* ^2^ with values ranging from 0% (homogeneity) to 100% (heterogeneity). To explain variance of depressive symptom reduction—the relation between study-level characteristics and the magnitude of the depression reduction effect size (*d*_+_)—a modified, weighted least squares regression was used with weights equal to the inverse variance of each exercise intervention effect size (viz., fixed-effects meta-regression). The underlying assumptions of meta-regression are similar to that of ordinary least-squares regression, including independence of errors, homoscedasticity of variance, and normally distributed variables [28], [33], [39], [40]. Statistically significant bivariate regression analyses were integrated into a multiple-moderator fixed effects regression to determine which variables could explain unique between study variance. To reduce multicollinearity in multiple meta-regression models, all continuous variables were zero centered based on their means; categorical variables were contrast coded (−1/+1). Beta-values (β) appear in standardized form in order to quantify the amount of variability in *d*s associated with each moderator of interest. All meta-regression model estimated effect sizes are depicted using the moving constant technique, entering multiple predictor variables simultaneously [41]. Two-sided statistical significance was *p*<0.05.

## Results

### Methodological Characteristics

**Figure 1:**
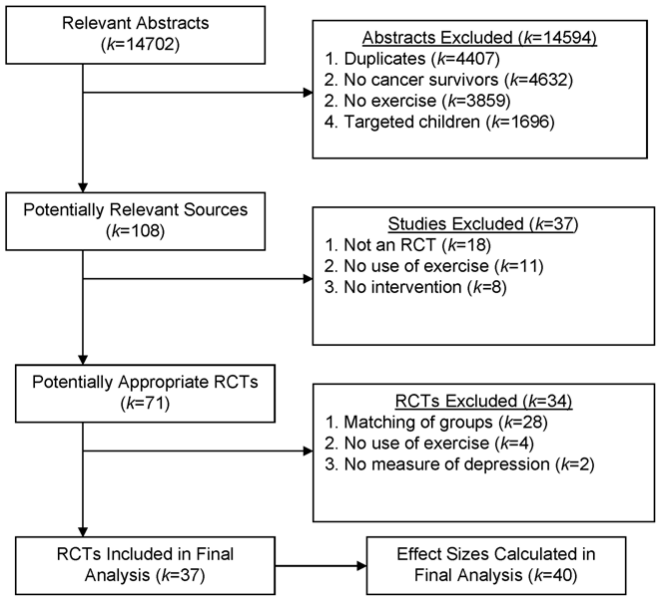
Flow diagram of exercise intervention identification and selection.

Qualifying for inclusion in the meta-analysis were 37 relevant randomized controlled exercise interventions [17]–[20], [42]–[74] (N = 2,929) with a total of 40 comparisons (*k* = 40) of exercise versus control conditions (Figure 1). Thirty-four studies provided one effect size, and three provided two effect sizes [20], [44], [52]. Exercise interventions were published in 2006±4.2 (range: 1994–2010) with most studies (70%) conducted in North America. The mean PEDro score of the exercise interventions was 7.0±1.0 suggesting relatively high methodological quality [22]. A minority of studies (20%) reported that they implemented at least one theory of behavior change (Table 1). Studies assessed depression using the Center for Epidemiologic Studies-Depression questionnaire (40%) [25], Profile of Mood States (23%) [23], the Beck Depression Inventory (18%) [24], Hospital Anxiety and Depression Scale (12%) [26], or Symptom Assessment Scale (7%) [27].

**Table 1:** Descriptive characteristics of included studies, subjects and exercise interventions by type of cancer (means ± SD, n(%), *k*(%) where noted).

Descriptive Statistic	All Cancer	Breast	Prostate	Leukemia	Lymphoma
**Study Characteristics**					
Number of studies, *k*	40^a^ (100%)	26^b^ (65%)	2 (5%)	2 (5%)	2 (5%)
Year of study	2006±4.2	2006±3.9	2008±2.1	2008±0.7	2006±3.5
Published in journal, *k*	34 (85%)	21 (81%)	2 (100%)	2 (100%)	2 (100%)
PEDro quality	7.0±1.0	6.7±1.1	7.0±0.0	7.5±0.7	7.5±0.7
**Subject Characteristics**					
Total n	2929 (100%)	1796 (61%)	121 (4%)	66 (2%)	161 (6%)
Gender, n of women	2548 (87%)	1796 (100%)	121 (0%)	22 (33%)	61 (38%)
Ethnicity, n (% total n)					
White, non-Hispanic	2255 (77%)	1437 (80%)	–	–	–
African-American	498 (17%)	296 (16%)	–	–	–
Hispanic	88 (3%)	54 (3%)	–	–	–
Asian	59 (2%)	18 (1%)	–	–	–
Age, yr	51.3±6.5	50.9±4.7	68.5±1.2	45.2±8.6	52.1±1.5
Stage of treatment, *k*					
Currently treated	29 (73%)	17 (65%)	2 (100%)	2 (100%)	2 (100%)
Previously treated	11 (27%)	9 (35%)	–	–	–
Time since diagnosis, mo	25.3±19.6	26.9±21.3	–	–	29.2±8.0
**Exercise Intervention Characteristics**					
Intervention length, wk	13.2±11.7	15.5±14.2	12.0±5.6	4.0±1.4	9.5±3.5
Length, min•session^−1^	49.1±27.1	54.7±27.5	65.0±35.4	36.0±33.9	61.2±40.6
Frequency, session•wk^−1^	3.0±2.5	2.8±1.3	2.0±1.4	5.0±0.0	2.0±1.4
Exercise volume, min•wk^−1^	123.9±52.2	135.2±25.1	105.0±21.2	180.0±169.7	97.5±0.0
Aerobic intensity, MET	4.8±1.1	4.7±0.9	4.4±0.8	5.4±2.3	7.0±0.0
Strength intensity, MET	2.9±0.5	2.9±0.6	3.0±0.0	3.0±0.0	2.5±0.0
Neuromuscular, MET	2.5±0.0	2.5±0.0	–	–	2.5±0.0
Flexibility, *k*					
Included	20 (50%)	13 (50%)	2 (100%)	1 (50%)	1 (50%)
Excluded	20 (50%)	13 (50%)	–	1 (50%)	1 (50%)
Supervision, *k*					
Supervised	24 (60%)	19 (73%)	2 (100%)	2 (100%)	2 (100%)
Unsupervised	16 (40%)	7 (27%)	–	–	–
Use of theory, *k*					
None	32 (80%)	21 (81%)	2 (100%)	2 (100%)	1 (50%)
Psychological	8 (20%)	5 (19%)	–	–	1 (50%)
Depression Scale used, *k*					
CES-D	16 (40%)	9 (35%)	1 (50%)	–	2 (100%)
POMS	9 (23%)	7 (27%)	–	1 (50%)	–
BDI	7 (18%)	6 (23%)	1 (50%)	–	–
HADS	5 (13%)	2 (8%)	–	1 (50%)	–
SAS	3 (8%)	2 (8%)	–	–	–

**NOTE:** Percentages may not sum to 100% due to rounding error.

CES-D, Center for Epidemiologic Studies Depression scale; POMS, Profile Of Mood States; BDI, Beck Depression Inventory; HADS, Hospital Anxiety and Depression Scale; SAS, Symptom Assessment Scale.

*k*, number of studies included.

MET, metabolic equivalent, 1MET = 3.5 ml O_2_·kg·min^−1^.

a 37 studies provided 40 total effect size estimates.

b 24 studies provided 26 total effect size estimates.

### Cancer Survivor Characteristics

Cancer survivors participating in the exercise trials averaged 51.3±6.5 yr (range: 39–70). The majority of cancer survivors participating in the exercise interventions were white, non-Hispanic (n = 2,255; 77%), and women (n = 2,548; 87%) with a time since cancer diagnosis of 25.3±19.6 months (range: 2.8–73.0). Exercise interventions were more common during curative therapy with 29 of the 40 exercise interventions (73%) occurring during treatment (i.e., chemotherapy or radiation treatment). Trials most often examined breast cancer survivors (*k* = 24) [17], [20], [48]–[69]. Two trials each focused on prostate cancer [19], [70], leukemia [72], [73], and lymphoma [18], [74] survivors, and only one trial examined colorectal cancer survivors [71]. The remaining 6 trials examined survivors with diverse types of cancer diagnoses [42]–[47]. At baseline, the standardized metric of depressive symptoms was 34.2±26.9 and ranged from 3.49 to 81.5.

### Exercise Intervention Characteristics

The mean length of the exercise interventions was 13.2±11.7 wk with an average of 3.0±2.5 sessions per week lasting 49.1±27.1 min·session^−1^. Average weekly volume of all exercise was 129.4±64.9 min·wk^−1^. Exercise modalities included walking (*k* = 16; 40%), stationary cycling (*k* = 5; 13%), weight machines (*k* = 2; 5%), resistance bands (*k* = 3; 8%), and yoga (*k* = 8; 20%). In addition, flexibility exercises were prescribed in 50% of the exercise interventions. The absolute intensity of exercise was 3.9±1.3 METs indicating they were of low (i.e., <3 METs) to moderate (i.e., ≥3 to <6 METs) intensity. A majority of exercise interventions (60%) was supervised. Table S1 summarizes methodological characteristics of the included trials.

### The Influence of Exercise on Depressive Symptoms

**Figure 2:**
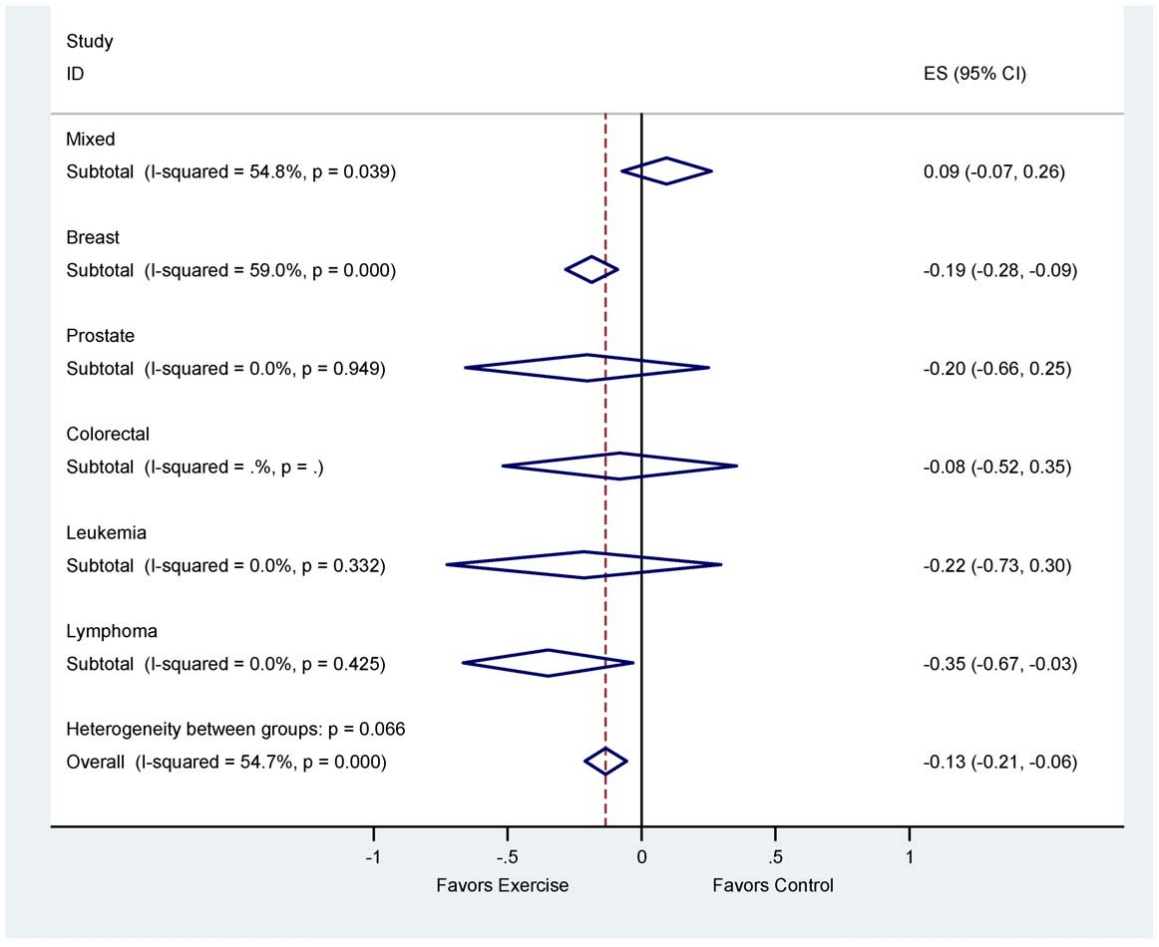
Forest plot of depressive symptom reduction by cancer group.

Exercise provided a small overall reduction in depressive symptoms compared to standard care among all types of cancer [*d_+_* = −0.13 (95% CI: −0.26, −0.01)]. Subgroup analysis by cancer type revealed significant reductions in depressive symptoms among breast cancer survivors [*d_+_* = −0.17 (95% CI: −0.32, −0.02)], but no significant difference in depressive symptoms among prostate, leukemia, lymphoma, and colorectal cancer survivors (Table 2). Figure 2 depicts the fixed-effects mean reduction in depressive symptoms, stratified by type of cancer. Collectively, the 40 effect sizes lacked homogeneity [*I* ^2^ = 55% (95% CI: 35–68), p<0.001], as did the analysis when restricted to breast cancer survivors [*I* ^2^ = 59% (95% CI: 37–73), p<0.001; Table 2].

**Table 2:** Weighted mean effect of exercise modulating depressive symptoms by type of cancer.

		*d*_+_ (95% CI)		Consistency of *d*s		
Type of Cancer	*k*	Fixed Effects	Random Effects	*Q* c	I^2^ (95% CI)	*P* c
**All Cancer**	40^a^	−0.13 (−0.21, −0.06)	−0.13 (−0.26, −0.01)	86.13	55% (35, 68)	<0.001
**Breast**	26^b^	−0.19 (−0.28, −0.09)	−0.17 (−0.32, −0.02)	60.79	59% (37, 73)	<0.001
**Prostate**	2	−0.20 (−0.66, 0.25)	−0.20 (−0.82, 0.40)	0.00	0% (0, 100)	0.948
**Leukemia**	2	−0.22 (−0.73, 0.30)	−0.24 (−0.89, 0.40)	0.94	0% (0, 100)	0.332
**Lymphoma**	2	−0.35 (−0.67, −0.03)	−0.30 (−0.89, 0.29)	0.64	0% (0, 100)	0.424
**Colorectal**	1	−0.08 (−0.52, 0.35)	–	–	–	–

**NOTE:** Weighted mean effect size values (*d_+_*) are negative when the exercise intervention was successful in reducing depression compare to standard care.

*k*, number of studies.

a 37 studies provided 40 total effect size estimates.

b 24 studies provided 26 total effect size estimates.

c Significance implies rejection of the hypothesis of homogeneity and the inference of heterogeneity.

### Moderators of the Influence of Exercise on Depressive Symptoms

Three moderators explained unique variance relating to the efficacy of exercise to reduce depressive symptoms when entered in a multiple meta-analysis regression model. Weekly volume of aerobic exercise reduced depression in dose-response fashion (β = −0.24, p = 0.03), a pattern that was only evident in higher quality trials. Exercise reduced depressive symptoms most when exercise sessions were supervised (β = −0.26, p = 0.01) and cancer survivors were between 47–62 yr [(β = 0.27, p = 0.01); Table 3]. These three moderators together explained 35% of the variance in depression reduction resulting from exercise; yet, variability beyond that expected by sampling error alone remained unexplained.

**Table 3:** Characteristics related to depressive symptoms change for all cancer survivors.

Study dimension and level^a^			Adjusted^b^ *d*_+_ (95% CI)	β^c^	P
**Accumulated weekly volume of aerobic exercise, min•wk^1^×PEDro methodological score** (Continuous )^d^	PEDro = 10 (higher quality)	90 min·wk^−1^	−0.07 (−0.42, 0.27)	−0.24^e^	0.03
		120 min·wk^−1^	−0.28 (−0.54, −0.02)		
		150 min·wk^−1^	−0.49 (−0.77, −0.23)		
		180 min·wk^−1^	−0.71 (−1.09, −0.33)		
	PEDro = 5 (lower quality)	90 min·wk^−1^	−0.29 (−0.54, 0.04)		
		120 min·wk^−1^	−0.19 (−0.40, 0.02)		
		150 min·wk^−1^	−0.09 (−0.34, 0.14)		
		180 min·wk^−1^	0.00 (−0.34, 0.34)		
**Supervision of exercise**		Unsupervised	−0.13 (−0.23, −0.04)	−0.26	0.01
		Supervised	−0.36 (−0.55, −0.18)		
**Age,** f **y** (Continuous -*Quadratic*)		40	0.16 (−0.08, 0.41)	0.27	0.01
		50	−0.20 (−0.30, −0.10)		
		60	−0.25 (−0.42, −0.08)		
		70	0.01 (−0.47, 0.56)		

**NOTE:** Weighted mean effect size values (*d_+_*) are negative when the exercise intervention reduced depression compared to the control group.

a Levels represent values of interest of each moderator; in these models, continuous variables were represented in their continuous form; the estimates adjust for the other moderators in the model.

b *d*_+_ and their 95% CI estimates statistically adjust for the presence of the rest of the moderators in the fixed-effects model, including weekly minutes of exercise×PEDro interaction and their independent linear terms, supervision of exercise, quadratic and linear trends for age, held constant at their means except for the study dimension in question.

c β values are standardized.

d This is a continuous×continuous interaction. We chose to report PEDro scores of 5 and 10 to highlight the variability along the continuous distribution of PEDro scores, those of very high quality (i.e., 10) versus those of low quality (i.e., 5).

e β for interaction. Independent β: weekly aerobic volume, β = −0.09; PEDro methodological score, β = −0.28.

f Continuous quadratic trend including linear component.

In our bivariate analyses (Table S2), three other features related to the magnitude of exercise-induced reduction of depressive symptoms: explicit use of theory in intervention development, the percentage of non-Hispanic Whites in the sample, and time since cancer diagnosis. Interventions were more successful in reducing depressive symptoms when they used psychological theory, sampled greater percentages of non-Hispanic Whites, and were more proximal to the date of cancer diagnosis. Still, none of these moderators explained significant variability in combined moderator analysis, suggesting that their influence is explained by the variables in the combined moderator model (viz., supervision, volume of exercise, methodological quality, and age; see Table 3). Standardized baseline depressive symptom scores were not associated with depressive symptom improvements resulting from exercise (p = 0.71).

## Discussion

This review found that exercise provided a small overall reduction in depressive symptoms among cancer survivors, *d_+_* = −0.13 (95% CI: −0.26, −0.01), but the amount of change varied widely across studies. Our analysis revealed exercise reduced depressive symptoms among breast cancer survivors, *d_+_* = −0.17 (95% CI: −0.32, −0.02), a pattern that confirms previous reports in the literature [2], [75], [76]. We observed non-significant exercise-related reductions in depressive symptoms among prostate, colorectal, leukemia, and lymphoma survivors, but the lack of statistical significance among these types may be due in part to the small numbers of included studies and subsequent lowered statistical power to detect effects (Table 2). Of note, in bivariate analyses a model related to type of cancer revealed no difference (results not shown), suggesting that the depression-reducing effects of exercise may generalize to other types of cancer.

Studies included in our analysis implemented an array of depression measures to indicate whether one may suffer from depression. The questionnaires used to assess depression varied with respect to content of questions, scoring, and cut-points used for clinical judgment, making the comparability of depression at baseline between trials difficult, and making the clinical generalizability of the current results more difficult. Therefore, we chose to focus our discussion on the standardized mean difference effect size, and the statistical interpretation of the association between exercise and depressive symptoms, rather than clinical significance.

We attempted to elucidate the exercise dose and clinical characteristics moderating the overall reduction of depressive symptoms among cancer survivors. To date, one meta-analysis has examined moderator variables associated with improvements in depressive symptoms among cancer survivors [76]. This previous meta-analysis examined individual moderators of depressive symptoms [76], whereas our meta-analysis examined multiple moderators simultaneously. Aerobic exercise reduced depressive symptoms in dose-response fashion such that as weekly minutes of aerobic exercise increased, so did reductions in depressive symptoms, a finding observed only in higher quality trials (Table 3). In higher quality trials, the amount of depressive symptom reduction reached large magnitude for those with 3 hours per week of aerobic exercise.

Since the overall mean reduction in depressive symptoms was small in magnitude, it is plausible that only the methodologically rigorous studies were able to detect such an effect in depressive symptom reduction. These trends are consistent with evidence suggesting exercise reduces depressive symptoms in dose-response fashion among populations with depression [77] and among cancer survivors [76]. Consistent with our findings, the American College of Sports Medicine consensus statement on exercise and cancer survivorship suggests all cancer survivors should strive to achieve a large volume of aerobic exercise of ≥150 min·wk^−1^ to maximize the health benefits [2]. Moreover, accumulating large volumes of aerobic exercise should be progressive, increasing duration and frequency of exercise over weeks or months of exercise training as the course of the disease process allow and functional capacity improves [2], [78], [79].

Our results showed that cancer survivors engaging in supervised exercise experienced less depressive symptoms than those who engaged in unsupervised exercise. Similar patterns have appeared in prior meta-analyses addressing the effects of exercise on quality of life [14], fatigue reduction [80], and depressive symptoms [76] among cancer survivors. Moreover, Spence *et al.*, and Whitehead *et al.*, found breast and colon cancer survivors prefer supervised exercise training over unsupervised exercise [81], [82].

We found exercise reduced depressive symptoms more among cancer survivors between 47–62 yr than those younger than 47 yr and older than 62 yr. Because previous studies reported higher levels of psychosocial stress, including depressive symptoms, among younger cancer survivors [83], [84], we hypothesized it would be younger cancer survivors who would experience the greatest reductions in depressive symptoms attributable to exercise. It is unclear why cancer survivors younger than 47 yr did not experience significant exercise-induced reductions in depressive symptoms, on average (Table 3). One possibility is that the average weekly aerobic exercise volume performed (∼130 min·wk^−1^) was not a large enough dose of exercise to reduce depressive symptoms among cancer survivors younger than 47 yr. The fact that no significant reduction in depressive symptoms among cancer survivors older than 62 yr appeared may be due in part to a floor effect [10]. That is, older cancer survivors report less depressive symptoms at baseline [85], and may show smaller exercise-induced improvements in depressive symptoms compared to those who are middle-aged.

The release of monoamine neurotransmitters (i.e., serotonin, dopamine, and norepinephrine) and endorphins during aerobic exercise has provided preliminary mechanistic support for the use of aerobic exercise to reduce and manage depressive symptoms [86], [87] and avoids common side-effects associated with anti-depressant medications [88], [89]. Interestingly, running distance is associated with improved neurological function; increasing neurotropic factors in the brain and improving mood [90]. Nonetheless, these hypotheses are limited in explaining the complex physiological and psychosocial etiologies of depressive symptoms [87]. Studies in the current meta-analysis rarely included physiological measures, impeding clear tests of such hypotheses. Continued research is necessary to examine mechanisms underpinning the reduction of depressive symptoms in response to exercise.

### Limitations

The major limitation of this meta-analysis is that depressive symptoms were a secondary outcome in almost all exercise interventions. As such, cancer survivors in the exercise trials that we meta-analyzed were not recruited based on depression levels; moreover, they may have exhibited few depressive symptoms at baseline. Nonetheless, our analysis suggests exercise is efficacious to improve depressive symptoms among cancer survivors. Our analysis may underestimate the efficacy of exercise to reduce depressive symptoms among cancer survivors with higher levels of depression or those with a diagnosis of depression. Furthermore, our analysis and interpretation of our findings have focused on the statistical associations, not the clinical implications of exercise and the experience of depressive symptoms.

Despite our intention to include all types of cancer of any race, 26 of the 40 effect sizes (65%) targeted white, non-Hispanic, breast cancer survivors exclusively, which has been a limitation of previous meta-analyses examining a variety of health-related outcomes among cancer survivors [14]–[16]. Additionally, the number of exercise interventions among breast cancer survivors limits the generalizability of our findings to other types of cancer, even though there was no significant difference in reduction of depressive symptoms attributable to type of cancer. These limitations should provide an impetus for researchers to continue investigating the effects of exercise among other ethnic groups and underrepresented cancer types.

Despite an overall rating of high methodological quality (7.0±1.0 of 11), we noted some consistent methodological weaknesses throughout the literature, such as inclusion of small sample sizes, inconsistent criteria with respect to study entry eligibility and baseline depressive symptoms levels, poor reporting of adverse events, and failure to follow intent-to-treat analytic strategies. Indeed, the dose-response pattern of exercise to the reduction of depression symptoms materialized most clearly only in the studies with the highest methodological quality.

### Conclusion

In closing, we confirmed that exercise provides a small overall reduction in depressive symptoms among cancer survivors. Depressive symptom reduction occurred in dose-response fashion with weekly volume of aerobic exercise, with high-quality trials documenting large changes for cancer survivors accruing larger weekly volumes of aerobic exercise. Larger reductions in depressive symptoms also occurred with supervised exercise, and among cancer survivors 47–62 yr. Cancer survivors should strive to avoid physical inactivity; discuss the safety and feasibility of exercising with their medical care provider to optimize depressive symptoms management; and eventually aim to achieve larger weekly volumes of aerobic exercise if possible, complimented with resistance training twice-weekly, and flexibility activity on days of non-exercise [2]. Furthermore, clinicians now have evidence to advocate the potential benefit of aerobic exercise as a modality to manage depressive symptoms among cancer survivors.

## Supporting Information

Table S1 Clinical, exercise, and methodological characteristics of included studies.
(DOCX)

Table S2 Bivariate moderator intervention characteristics related to depressive symptoms reduction for all cancer survivors.
(DOCX)
