# Supplementary material for: The Efficacy of Exercise in Reducing Depressive Symptoms among Cancer Survivors: A Meta-Analysis
Source: PLoS One. 2012 Jan 27;7(1):e30955. doi: 10.1371/journal.pone.0030955 (PMC3267760; doi:10.1371/journal.pone.0030955)
Supplement: Table S1 — Clinical, exercise, and methodological characteristics of included studies. (DOCX) [file pone.0030955.s001.docx]

**Table S1.** Clinical, exercise and methodological characteristics of included studies.

| **First Author, Year, Reference** | **Clinical Characteristics** | |  | **Exercise Characteristics** | | | | |  | **Methodological Characteristics** | |
| --- | --- | --- | --- | --- | --- | --- | --- | --- | --- | --- | --- |
|  | **Sample Size** | **Type of Cancer** |  | **Freq (d∙wk^-1^)** | **Intensity** | **Time (min∙ session^-1^)** | **Type** | **Duration (wk)** |  | **Depression Measure** | **PEDro Score** |
| **Mixed Cancer Diagnoses** | | | | | | | | | | | |
| **Burnham, 2002,**[42] | I = 12; C = 6 | Breast; Colon |  | 3 | 40‒60% HRR | 30 | Aerobic; treadmill, stationary cycle, stair climber | 10 |  | LASA | 7 |
| **Dimeo, 1999,**[43] | I = 27; C=32 | Variety; solid tumors, lymphoma |  | 7 | 50% HRR | 30 | Aerobic; supine biking ergometer | 4 |  | POMS | 8 |
| **Dodd, 2010,**[44] | I = 44 ;I (Delayed) = 36; C = 39 | Breast; Colorectal; Ovarian |  | 3‒5 | 60‒80% V0_2peak_ | 20‒30 | Aerobic; walking, jogging, bicycling | 52 |  | CES-D | 8 |
| **Berglund, 1994,**[45] | I = 98; C = 101 | Majority Breast cancer |  | 2 | n/a | 60 | n/a | 7 |  | HADS | 7 |
| **Courneya, 2003,**[46] | I = 60; C = 48 | Breast; Colon; Ovarian; Stomach; Melanoma; Hodgkin; Non-Hodgkin’s; Brain; Lung |  | 3‒5 | 65‒75% HR_max_ | 20‒30 | Aerobic; swimming, cycling | 10 |  | CES-D | 8 |
| **Thorsen, 2005,**[47] | I = 59; C = 52 | Lymphoma; Breast; Gyne-cologic; Testicular |  | 2 | 60‒70% HR_max_ | 30 | Aerobic; walking, cycling, jogging | 14 |  | HADS | 8 |
| **Breast Cancer** | | | | | | | | | | | |
| **Daley, 2007,**[17] | I = 34; C = 36 | Breast |  | 3 | 65‒85% HR_max_ | 50 | n/a | 8 |  | BDI | 8 |
| **Courneya, 2007,**[20] | I (Aer) = 78; I (RET) = 82; C = 82 | Breast |  | 3 | 60‒70% V0_2max;_ 2 Sets, 60‒70% predicted 1-RM | 15‒45 | Aerobic: cycle ergometer, treadmill, elliptical; 9 strength exercise | 17 |  | CES-D | 7 |
| **Culos-Reed, 2006,**[48] | I = 20; C = 18 | Majority breast |  | 1 | n/a | 75 | Yoga | 7 |  | POMS | 7 |
| **Rausch, 2007,**[49] | I = 15; C = 8 | Breast |  | 1 | n/a | 90 | Tai Chi | 10 |  | POMS | 5 |
| **Ohira, 2006,**[50] | I = 43; C = 43 | Breast |  | 2 | Progressive resistance | 60 | Weight training | 24 |  | CES-D | 6 |
| **Perna, 2010,**[51] | I = 26; C = 25 | Breast |  | 3 | Aerobic: 50‒85% HR_max_; Weight training: 1 set, 12‒15 reps | 30 | Aerobic; treadmill walking; Weight training; weight belts | 12 |  | CES-D | 9 |
| **Lee, 2010,**[52] | I = 16; C = 18 | Breast |  | 1 | Light (<40% 1-RM), elastic band, medicine ball exercise | 40 | Weight training of shoulder muscle groups | 8 |  | BDI | 5 |
| **Demark-Wahnefried, 2008,**[53] | I = 26; C = 29 | Breast |  | ≥3 | Aerobic: walking  Weight training: Light (<40% 1-RM) | ≥30 | Aerobic training; walking; Weight training: elastic band, medicine ball exercise | 12 |  | HADS | 6 |
| **Targ, 2002,**[54] | I = 74; C = 60 | Breast |  | 1 | n/a | 90 | Yoga | 12 |  | POMS | 6 |
| **Mutrie, 2007,**[55] | I =101; C =102 | Breast |  | 3 | 50‒75% HR_max_ | 45 | Walking, cycling, aerobics, strength exercises | 12 |  | BDI | 9 |
| **Latka, 2009,**[56] | I = 37; C = 38 | Breast |  | 5 | 60‒80% HR_max_ | 30 | Walking | 24 |  | CES-D | 7 |
| **Patel, 2004,**[57] | I = 43; C = 19 | Breast |  | 1 | n/a | 90 | Yoga | 12 |  | POMS | 6 |
| **Vadiraja, 2009,**[58] | I = 44; C = 44 | Breast |  | 3 | n/a | 60 | Yoga | 6 |  | HADS | 8 |
| **McClure, 2010,**[59] | I = 16; C = 16 | Breast |  | 7 | Low‒moderate intensity | 17 | Arm flexibility exercise | 17 |  | BDI | 6 |
| **Pinto, 2003,**[60] | I = 12; C = 12 | Breast |  | 3 | 60‒70% HR_max_ | 50 | Treadmill walking, arm cycling, stationary cycling, rowing | 12 |  | POMS | 7 |
| **Mock, 1997,**[61] | I = 22; C = 24 | Breast |  | 4‒5 | Self-paced | 20‒30 | Walking | 6 |  | SAS | 7 |
| **Danhauer, 2009,**[62] | I = 13; C = 14 | Breast |  | 1 | n/a | 75 | Yoga | 10 |  | CES-D | 6 |
| **Cadmus, 2009,**[63]**; (IMPACT)** | I = 25; C =25 | Breast |  | 5 | 60‒80% HR_max_ | 30 | Not-specified | 24 |  | CES-D | 8 |
| **Drouin, 2005,**[64] | I = 13; C = 8 | Breast |  | 5 | 50‒70% HR_max_ | 20‒45 | Treadmill walking | 7 |  | POMS | 7 |
| **Chandwani, 2010,**[65] | I = 30; C = 31 | Breast |  | 2 | n/a | 60 | Yoga | 6 |  | CES-D | 6 |
| **Vito, 2007,**[66] | I =13; C = 12 | Breast |  | 2 | n/a | 90 | Yoga | 8 |  | POMS | 8 |
| **Payne, 2008,**[67] | I =10; C = 10 | Breast |  | 4 | Moderate intensity | 20 | Walking | 12 |  | CES-D | 7 |
| **Mock, 1994,**[68] | I = 9; C = 9 | Breast |  | ≥3 | Self-paced | 30 | Walking | 6 |  | SAS | 7 |
| **Eyigor, 2010,**[69] | I = 27; C = 25 | Breast |  | 3 | n/a | 20‒30 | Pilates | 8 |  | BDI | 5 |
| **Prostate Cancer** | | | | | | | | | | | |
| **Culos-Reed, 2010,**[19] | I = 53; C = 47 | Prostate |  | 3‒5 | moderate | 60 | Walking, resistance exercise | 16 |  | CES-D | 7 |
| **Monga, 2007,**[70] | I = 11; C = 10 | Prostate |  | 3 | 65% HR_reserve_ | 30 | Treadmill walking | 8 |  | BDI | 7 |
| **Leukemia** | | | | | | | | | | | |
| **Jarden, 2009,**[72] | I = 21; C = 21 | Leukemia |  | 1 | 50‒75% HR_max_  1‒2 sets, 10‒12 reps | 60 | Aerobic: Stationary cycling; Resistance: Free weights, ankle weights | 4‒6 |  | HADS | 8 |
| **Chang, 2008,**[73] | I = 11; C = 11 | Leukemia |  | 5 | 60‒110 bpm | 12 | Walking | 3 |  | POMS | 7 |
| **Lymphoma** | | | | | | | | | | | |
| **Courneya, 2009,**[18] | I = 60; C = 62 | Lymphoma |  | 3 | 50‒75% V0_2peak_ | 20‒45 | Recumbent cycle ergometer | 12 |  | CES-D | 8 |
| **Cohen, 2004,**[74] | I = 20; C = 19 | Lymphoma |  | 1 | n/a | 60 | Yoga | 7 |  | CES-D | 7 |
| **Colorectal Cancer** | | | | | | | | | | | |
| **Courneya, 2003,**[71] | I = 69; C = 33 | Colorectal |  | 3‒5 | 65‒75% HR_max_ | 20‒30 | Walking | 16 |  | CES-D | 9 |

NOTE: I, Intervention (exercise group); C, control group

CES-D, Center for Epidemiologic Studies Depression scale; POMS, Profile Of Mood States; BDI, Beck Depression Inventory; HADS, Hospital Anxiety and Depression Scale; SAS, Social Anxiety Scale.

HR_max_, maximum heart rate; HRR, heart rate reserve; V0_2peak_, maximal oxygen consumption (ml∙kg∙min^-1^); bpm, beats per minute; 1-RM, one-repetition maximum
