# Supplementary material for: The Efficacy of Exercise in Reducing Depressive Symptoms among Cancer Survivors: A Meta-Analysis
Source: PLoS One. 2012 Jan 27;7(1):e30955. doi: 10.1371/journal.pone.0030955 (PMC3267760; doi:10.1371/journal.pone.0030955)
Supplement: Table S2 — Bivariate moderator intervention characteristics related to depressive symptoms reduction for all cancer survivors. (DOCX) [file pone.0030955.s002.docx]

**Table S2.** Bivariate moderator intervention characteristics related to depressive symptom reduction for all cancer survivors**.**

| **Study dimension and level^a^** | | ***d*_+_ (95% *CI*)^c^** | **β^d^** | **P** |
| --- | --- | --- | --- | --- |
| **Theory** | None | -0.06 (-0.15, 0.03) | 0.26 | 0.01 |
|  | Psychological | -0.26 (-0.39, -0.13) |  |  |
| **Supervision of exercise** | Supervised | -0.22 (-0.32, -0.13) | -0.37 | 0.002 |
|  | Non-supervised | 0.07 (-0.06, 0.21) |  |  |
| **Non-Hispanic white, %** | 24 | 0.02 (-0.21, 0.25) | 0.06 | 0.01 |
|  | 99 | -0.17 (-0.30, -0.05) |  |  |
| **Time since diagnosis, mo** | 2.8 | -0.17 (-0.29, -0.04) | 0.35 | 0.02 |
|  | 73.0 | 0.39 (-0.21, 1.00) |  |  |
| **Accumulated weekly volume of aerobic exercise, min∙wk^-1^** $\boldsymbol{\times}$ **PEDro methodological score** | PEDro = 5 × 90 min∙wk^-1^ | -0.19 (-0.43, 0.06) | -0.25 | 0.03 |
|  | PEDro = 5 × 150 min∙wk^-1^ | -0.02 (-0.26, 0.22) |  |  |
|  | PEDro = 10 × 90 min∙wk^-1^ | 0.05 (-0.24, 0.35) |  |  |
|  | PEDro = 10 × 150 min∙wk^-1^ | -0.35 (-0.61, -0.08) |  |  |
| **Age^e^, y** | 39 | 0.22 (-0.04, 0.47) | 0.70 | 0.001 |
|  | 51 | -0.19 (-0.27, -0.10) |  |  |
|  | 69 | 0.12 (-0.31, 0.54) |  |  |

NOTE: Weighted mean effect size values (*d_+_*) are negative when the exercise intervention was successful in reducing depression compared to the control group.

^a^Levels represent values of interest of each moderator. Values presented for the variables non-Hispanic white, time since diagnosis, accumulated weekly volume of aerobic exercise X PEDro methodological score, and age are point estimates from continuous variables along the regression line to better depict the pattern of results.

^b^*k*, For categorical variables, *k* denotes number of effect sizes in each group. For continuous variables, *k* denotes

total observations.

^c^bivariate *d*_+_ (95% *CI*) were calculated using fixed-effects models.

^d^β values are standardized.

**^e^**Quadratic trend including linear component.
